# Novel Use of a Smartphone to Measure Standing Balance

**DOI:** 10.2196/rehab.4511

**Published:** 2016-03-29

**Authors:** Nirtal Shah, Rosanne Aleong, Ivan So

**Affiliations:** ^1^David L. MacIntosh Sport Medicine ClinicFaculty of Kinesiology and Physical EducationUniversity of TorontoToronto, ONCanada; ^2^Rotman Research InstituteBaycrestToronto, ONCanada; ^3^Department of Electrical and Computer EngineeringUniversity of TorontoToronto, ONCanada

**Keywords:** mobile application, telemedicine, postural balance, sports medicine, mobile phone

## Abstract

**Background:**

Balance assessment and training is utilized by clinicians and their patients to measure and improve balance. There is, however, little consistency in terms of how clinicians, researchers, and patients measure standing balance. Utilizing the inherent sensors in every smartphone, a mobile application was developed to provide a method of objectively measuring standing balance.

**Objective:**

We aimed to determine if a mobile phone application, which utilizes the phone’s accelerometer, can quantify standing balance.

**Methods:**

Three smartphones were positioned simultaneously above the participants’ malleolus and patella and at the level of the umbilicus. Once secured, the myAnkle application was initiated to measure acceleration. Forty-eight participants completed 8 different balance exercises separately for the right and left legs. Accelerometer readings were obtained from each mobile phone and mean acceleration was calculated for each exercise at each ankle and knee and the torso.

**Results:**

Mean acceleration vector magnitude was reciprocally transformed to address skewness in the data distribution. Repeated measures ANOVAs were completed using the transformed data. A significant 2-way interaction was revealed between exercise condition and the body position of the phone (*P*<.001). Post-hoc tests indicated higher acceleration vector magnitude for exercises of greater difficulty. ANOVAs at each body position were conducted to examine the effect of exercise. The results revealed the knee as the location most sensitive for the detection of differences in acceleration between exercises. The accelerometer ranking of exercise difficulty showed high agreement with expert clinical rater rankings (kappa statistic>0.9).

**Conclusions:**

The myAnkle application revealed significantly greater acceleration magnitude for exercises of greater difficulty. Positioning of the mobile phone at the knee proved to be the most sensitive to changes in accelerometer values due to exercise difficulty. Application validity was shown through comparison with clinical raters. As such, the myAnkle app has utility as a measurement tool for standing balance.

## Introduction

Balance is defined as the body’s ability to remain steady with minimal sway and with the center of gravity over a base of support [1,2]. Balance is defined as the body’s ability not to fall by integrating the activities of the vestibular, somatosensory, visual, and musculoskeletal systems [1,3]. Improving standing balance has become a critical component of rehabilitation and has gained much attention in sports medicine as both preventive of and restorative for lower limb injuries. Balance training is utilized for injury prevention, performance enhancement, and for rehabilitation from sport-related and physical-activity-related injuries [1,4,5]. There is, however, little consistency in terms of how clinicians, researchers, and patients measure standing balance [1,6,7].

One commonly used method of assessing standing balance in sports medicine is the Balance Error Scoring System (BESS). The BESS shows inter-rater reliability ranging from poor to good, depending on how the evaluators were trained to score the BESS [8,9], as well as poor validity to detect subtle balance differences [8]. To improve reliability and validity, force plates are used in research to measure standing balance [10,11]. Force plates do not rely on clinical scoring and they have high test-retest reliability [12,13]. However, force plates are generally expensive and inaccessible to most clinicians and patients [6,14].

As a cost-effective and more accessible alternative, accelerometers are used to measure standing balance in athletes and nonathletes [14-16]. An accelerometer can be worn on the body to quantify human movement [14] by measuring movement in the x, y, and z axes. Body-worn accelerometers have demonstrated concurrent validity and high test-retest reliability when compared to force plates [14-16]. Some researchers have suggested that due to their portability, low-cost, and their ability to quantify human movement in natural and clinical environments, accelerometers are superior to force plates for quantifying standing balance [14-17].

Given patients’ poor accessibility to and understanding of current measures of balance, it is not entirely surprising that these measures are ignored by individuals undergoing treatment for impaired balance. Additionally, the literature points to poor patient compliance with rehabilitation exercises [18]. Perhaps increasing patients’ understanding of what is being measured and their ability to track their own progress would address part of the compliance issue. As technology advances, devices such as accelerometers become more affordable and more widely available. In fact, every smartphone contains an accelerometer. As such, the authors have designed a mobile phone app, myAnkle, that utilizes the built-in accelerometer to quantify standing balance.

The aims of the current study are to: (1) determine whether the app can distinguish between balance exercises of varying difficulty on a firm or foam surface; (2) compare various bodily locations for the mobile phone and their effects on measuring standing balance; and (3) assess the validity of the myAnkle app, as rated by expert clinical raters.

## Methods

The study protocol was approved by the Office of Research Ethics at the University of Toronto. Participants were recruited using posters within an athletic facility and a sports medicine clinic at a university, by verbally soliciting interested participants from varsity teams and, subsequently, by snowball sampling.

### Subjects

Fifty participants were recruited for the study. Two participants halted testing of their own accord due to fear of injury; ultimately, 48 participants completed the full testing protocol. Reported results reflect the 48 participants who completed the full protocol. All participants were between 18-30 years old (mean = 22 years; SD = 2.5 years). Twenty-one males and 27 females were tested with the myAnkle app on 8 different balance conditions separately for the right and left lower limbs (Table 1).

All participants were free of ankle, hip, knee, and lower-back injuries at the time of testing and had not sustained any injuries for at least 1 month prior to testing based on self-report. Demographic data for participants included in the analyses are summarized in Table 2. Informed consent was obtained from all participants prior to testing.

**Table 1:** Balance conditions (in the order they were tested).

Balance Condition	Eyes Open	Surface
Double leg stance, feet apart	Yes	Ground
Double leg stance, feet together	Yes	Ground
Tandem (1 foot in front of the other)	Yes	Ground
Single leg stance	Yes	Ground
Single leg stance	No	Ground
Double leg stance, feet apart	Yes	Foam Board
Single leg stance	Yes	Foam Board
Single leg stance	No	Foam Board

**Table 2:** Demographics.

Characteristic		
Age, y, mean (SD)		22 (2.5)
Height, cm, mean (SD)		175 (9.7)
Weight, kg, mean (SD)		72.57 (1.29)
Physical activity^a^, d, mean (SD)		5.08 (1.2)
Physical activity^a^, hr, mean (SD)		11.33 (5.5)
Gender	Male	21
	Female	27
Team	Varsity	33
	Nonvarsity	15
Handedness	Right	43
	Left	4
	Both	1
Kicking foot	Right	45
	Left	3

^a^Self-reported average days or hours of physical activity over the past year over 7 days.

### Exercises

Eight balance exercises were selected representing a gradation of exercise difficulty including firm or foam surfaces and with the participants’ eyes open or closed (Table 1). Exercise selection was based upon a review of the literature and a pilot study we performed with 8 subjects**.**

### Clinical Assessment of Exercise Difficulty

To obtain clinical rankings of the 8 balance exercises, 8 clinicians (3 physical therapists and 5 athletic therapists) were asked to rank the exercises from easiest to hardest. The clinicians were blinded to the results of the study and had an average of 12 years of clinical experience working in a sports medicine environment. Each clinician was provided with 1 full-page picture of each balance condition as well as the foam surface used in the testing and asked to rank the exercises using their clinical experience.

### Testing Protocol

**Figure 1:**
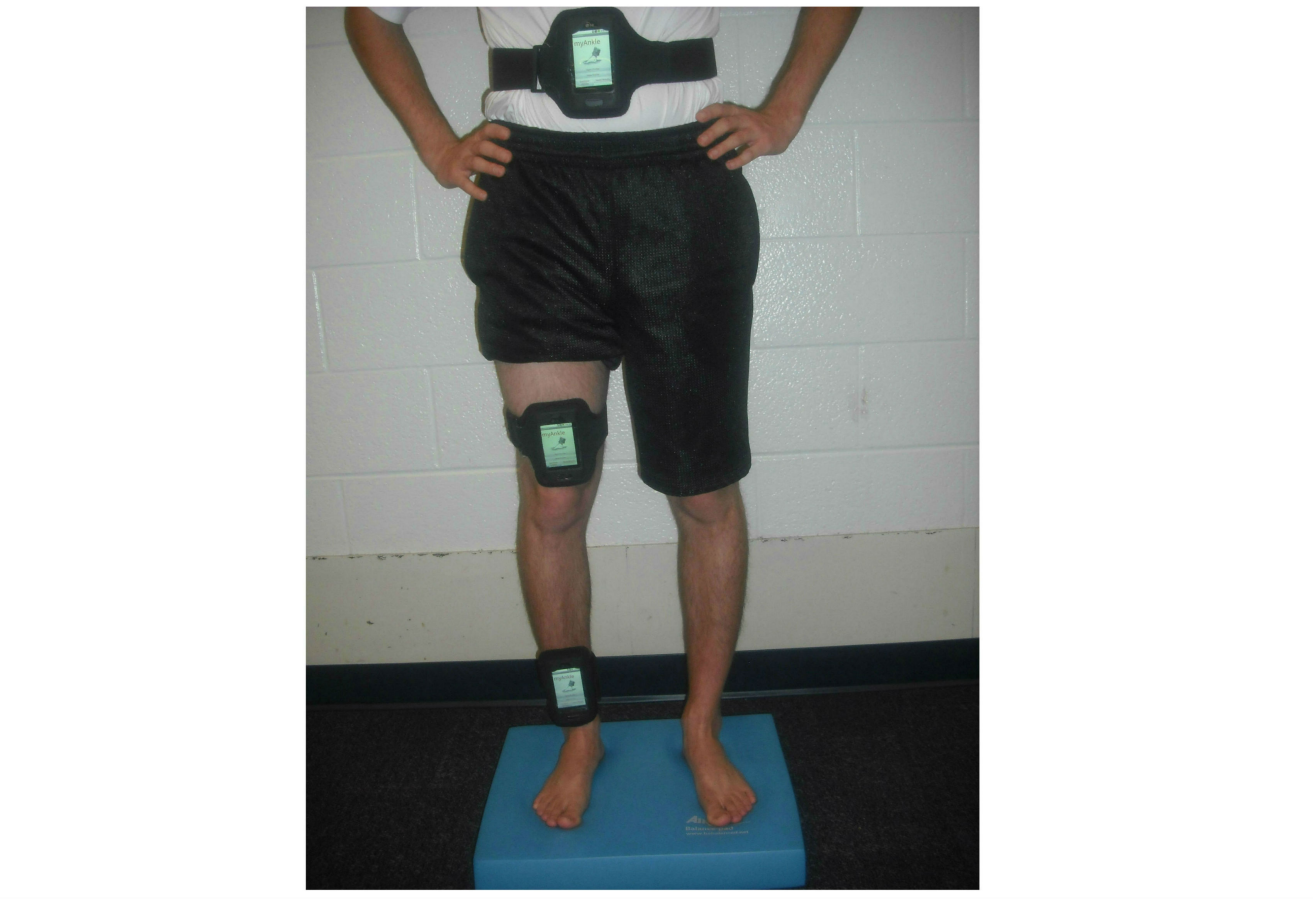
This picture shows the position of the mobile phones, participant testing position (hands on the hips), and the foam board used for balance testing. Three identical mobile phones were utilized. The first phone was positioned so that its lower edge was above the talocrural joint line, the second so its lower edge was above the superior midline of the patella, and the last so its center was at the level of the subjectÃ¢â‚¬â„¢s umbilicus. All subjects wore shorts and a t-shirt with no shoes or socks, as depicted.

All testing was completed by the primary author. Three identical mobile phones were strapped to the participant’s body using a commercially available, Velcro arm band for mobile phones (Figure 1). Additional Velcro was sewn onto the armband to extend the circumference such that it would accommodate the mobile phone being strapped above the knee and around the umbilicus.

No participant had prior knowledge of the testing protocol and each was given verbal instructions prior to each balance exercise. For all of the “eyes open” conditions, the participant was instructed to fixate on a black “X” located on a wall 437 cm from the participant. Participants were counterbalanced for the starting leg of testing. The rest periods between balance exercises and between right and left legs were 30 seconds and 3 minutes, respectively.

### Accelerometer

The LG Optimus One (P500h), which utilizes Google’s mobile platform Android 2.2 (also known as Froyo), was utilized for all testing. The mobile phone weighed 129 grams and its dimensions were 113.5 mm long, by 59 mm wide, by 13.3 mm deep. The mobile phone accelerometer was triaxial, measuring acceleration in the x, y, and z axes. The accelerometer sampled at a rate of 14-15 Hz, the result of hardware limitations. Consequently, between 420-450 raw measurements were collected for each exercise on each of the 3 axes.

### Calibrating the Accelerometer

Each of the 3 mobile phones was calibrated once at the start of the study. The purpose of the calibration was to adjust for any static bias of the accelerometer. Static bias is the inaccuracy in the accelerometer readings when the phone is immobile. The accelerometer measures in both the positive and negative directions for all 3 axes (ie, x+, x−, y+, y−, z+, z−). The static bias affects each of the 6 directions independently to varying degrees. Each phone was calibrated using the following procedure:

1. The mobile phone was placed on a stable, level surface so that 1 of the axes was vertical.
2. Accelerometer measurements were recorded for 30 seconds.
3. Steps 1 and 2 were repeated for all 6 directions (ie, for each side of the mobile phone).

A correction factor (C) for each direction for each phone was obtained by dividing the acceleration due to the force of gravity (9.81 m/s^2^) by the mean of all the samples in a given axis plane, both positively and negatively. For example, if the mean of all samples in the x+ direction was 9.95, then Cx+ = 9.81 / 9.95 = 0.986. A total of 6 correction factors were derived for each phone. The specific correction factors for a given phone were applied to each of the 420-450 raw measurements collected by that phone for each exercise performed.

### Mean R Calculation

Each accelerometer measurement comprises 3 values, 1 for each axis (ie, x, y, and z). The values were first corrected for static bias using the correction factor as described above. For example, if the raw x-axis value (x_raw) of a measurement was positive, the corrected value (x_corr) would be x_corr = x_raw × Cx+. Likewise, if the value was negative, then x_corr = x_raw × Cx−. Once the corrected value was applied, the magnitude of the resultant vector (R) was calculated for each of the 420-450 measurements as sqrt(x_corr^2^+ y_corr^2^+ z_corr^2^). The metric mean R is the average magnitude of the 420-450 resultant vectors. The mean R variable was then used as an index of balance for all subsequent analyses. A Perl script was used to calibrate, correct, and calculate all mean R values.

### Statistical Analyses

In order to assess the consistency of clinicians’ ratings of exercise difficulty, pairwise weighted kappa statistics were calculated for each combination of rater pairs. Further, pairwise kappa statistics were calculated between each clinical rater and the accelerometer device, providing a measure of device validity.

The calculated mean R variable described above was used in all subsequent statistical analyses. The observed values were initially assessed with respect to their distribution. In the event of a non-Gaussian distribution, various data transformations were utilized and characterized. Repeated measures analysis of variance (ANOVA) was then conducted with 3 within-subject variables (eg, exercise condition, leg tested, body position of phone) and 1 co-variate (eg, participant’s sex). Post-hoc comparisons with Bonferroni correction for multiple comparisons were then conducted to tease apart any interactions.

## Results

### Distribution of Mean R Data

Data were collected and classified according to exercise condition, leg tested, and body position of phone. With 8 exercise conditions, 2 legs tested, and 3 body positions for the phones, this resulted in a total of 48 individual cells of data. Examination of the calibrated and corrected mean R data revealed a non-Gaussian distribution for the majority of the 48 cells. A reciprocal transformation (1/mean R) was then implemented for all observations. The reciprocal mean R values represent the time required in seconds to reach a given velocity.

The reciprocal transformation rendered the distribution for most of the cells reasonably bell-shaped but 2 cells continued to exhibit bimodal distributions. The 2 cells with bimodal distributions were examined in further detail. The data were separated into 2 groups via a median split. The data were then examined for group differences with regards to the demographic (eg, sex, handedness, foot dominance), anthropometric (eg, foot length), and clinical measures (eg, number of injuries, physical activity) collected. Chi-square analyses for both cells revealed a significant effect of participant sex (λ^2^(1,48) = 6.857 (*P*<.01); λ^2^(1,48) = 30.561 (*P*<.001)). With participant sex used as a covariate in subsequent ANOVAs, residual distributions appeared bell-shaped.

### Independent Clinical Assessment of Exercise Difficulty

Using an 8-point scale where 1 is least difficult and 8 is most difficult, 8 independent therapists individually ranked the 8 balance conditions based upon their clinical experience of each condition’s difficulty (Table 3). Pairwise weighted kappa statistics were calculated for all combinations of the therapists for a total of 28 values ranging from 0.88 to 0.98, indicating high accordance between raters.

The myAnkle app rankings of exercise difficulty were identical for each body location (ie, ankle, knee, torso). Pairwise kappa statistics assessing concordance between the device ranking of exercise difficulty and expert rankings revealed values ranging from 0.9 to 1.0. With all kappa values above 0.8, this was interpreted as good agreement between the device and clinical experts [19].

**Table 3:** Therapist and myAnkle difficulty ranking of the 8 balance conditions.

Assessor	Difficulty Ranking							
	1	2	3	4	5	6	7	8
Therapist A	DL^a^	FT^b^	TAN^c^	SLS^d^	FDL^e^	SLSE^f^	FSLS^g^	FSLSE^h^
Therapist B	DL	FT	SLS	FDL	TAN	FSLS	SLSE	FSLSE
Therapist C	DL	FT	TAN	SLS	FDL	FSLS	SLSE	FSLSE
Therapist D	DL	FT	FDL	TAN	SLS	FSLS	SLSE	FSLSE
Therapist E	DL	FT	TAN	FDL	SLS	FSLS	SLSE	FSLSE
Therapist F	DL	FT	FDL	SLS	TAN	SLSE	FSLS	FSLSE
Therapist G	DL	FT	TAN	FDL	SLS	SLSE	FSLS	FSLSE
Therapist H	DL	FT	FDL	TAN	SLS	SLSE	FSLS	FSLSE
myAnkle (R^i^)	DL (8.0)	FT (6.9)	FDL (6.0)	TAN (4.6)	SLS (2.5)	SLSE (1.2)	FSLS (0.9)	FSLSE (0.5)

^a^DL: double leg stance on ground, feet apart

^b^FT: double leg stance on ground, feet together

^c^TAN: tandem (1 foot in front of the other)

^d^SLS: single leg stance on ground

^e^FDL: double leg stance on foam, feet apart

^f^SLSE: single leg stance on ground, eyes closed

^g^FSLS: single leg stance on foam

^h^FSLSE: single leg stance on foam, eyes closed

^i^Mean reciprocal R values for the myAnkle app at the knee location.

### Effects of Exercise Condition, Leg Tested, and Body Position of the Mobile Phone

A repeated measures ANOVA was conducted on reciprocal acceleration values with 3 within-subject variables (eg, exercise condition, leg tested, body position of phone) and 1 co-variate (eg, participant’s sex). A significant 3-way interaction was revealed between exercise condition, leg tested, and body position of the phone (F(14,644) = 19.490, *P*<.001).

As noted in the introduction, our primary questions of interest included: (1) whether myAnkle is capable of differentiating balance performance on exercises of varying difficulty and (2) at which body location should the mobile device be positioned for maximal detection. In light of our research foci and the identified 3-way interaction, we first explored the issue of laterality by examining the laterality-exercise interaction at each body location. A significant interaction was found for the ankle (F(7322) = 13.021, *P*<.001) and knee locations only (F(7322) = 16.098, *P*<.001). The effect of exercise at each leg-body position combination (ie, right leg-torso, left leg-torso, right leg-knee, left leg-knee, right leg-ankle, left leg-ankle) from separate, univariate ANOVAs with 1 within-subject variable (eg, exercise condition) and participant’s sex as a covariate reflects this finding also. Table 4 presents exercise and residual sums of squares for each ANOVA.

The exercise sum of squares values provide an estimate of variation due to different types of exercise while residual sum of squares values provide an estimate of unexplained variation. Residual variability for the ankle location was higher than that of either the knee or torso, indicating the ankle to be a less ideal location for the smartphone.

In the case of the more difficult exercises (ie, single leg stance on ground, eyes closed; single leg stance on foam; single leg stance on foam, eyes closed), higher raw acceleration values were observed. As a consequence of the reciprocal transformation (1/mean R), any potential differences in detection sensitivity between the different body locations for these more difficult exercises would be obscured.

Thus, separate, univariate ANOVA were completed including only these 3 exercises with 1 within-subject variable (eg, exercise condition) and participant’s sex as a covariate for each leg-body position combination (ie, right leg-torso, left leg-torso, right leg-knee, left leg-knee, right leg-ankle, left leg-ankle). Reciprocally transformed data were used due to a non-Gaussian distribution of the raw data for these 3 exercises. The general finding was that the exercise sums of squares values for the ankle and knee locations were comparable; however, the residual sums of squares values for the ankle location were slightly lower than those of the knee but at the cost of possibly increased asymmetry between the left and right ankle and the practical cost of necessitating a move of the mobile phone during testing. Refer to Table 5 for detailed results.

**Figure 2:**
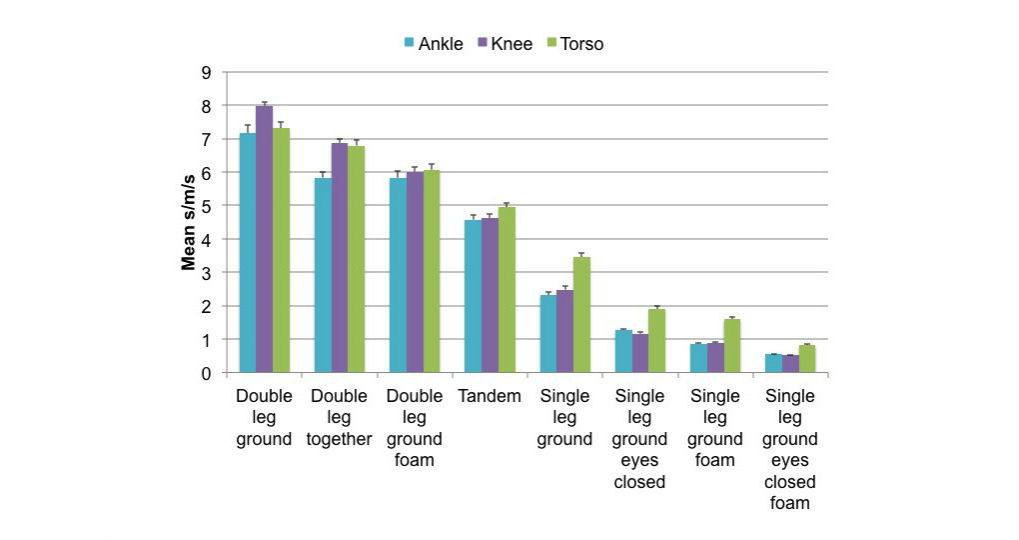
Mean reciprocal R values. A significant 2-way interaction between exercise condition and body position (P<.001) was identified. Mean reciprocal R values and standard error of the mean values are reported for each exercise condition for each body position.

The repeated measures ANOVA also revealed a significant 2-way interaction of exercise condition by body position (F(14,644) = 14.151, *P*<.001). In post-hoc analyses of this interaction, we see a significant effect of exercise at all 3 body position locations (*P*<.001); the largest effect of exercise was at the knee. Based upon these findings, we contend that the knee appears to be the best location at which to position the mobile phone to detect differences in balance performance across exercises. Refer to Figure 2 for the mean values.

In summary, we find that the mobile app can distinguish between exercises of varying difficulty and that the knee location overall maximally detects balance differences.

**Table 4:** Sums of squares for univariate ANOVA models—exercise condition and residual—at each body location (*P*<.001 for all locations).

Leg-Body Position	Sum of Squares Exercise	Sum of Squares Residual
Right leg-torso	754.168	237.958
Left leg-torso	785.728	259.915
Right leg-knee	1062.591	159.272
Left leg-knee	1183.151	178.214
Right leg-ankle	767.891	337.189
Left leg-ankle	621.819	265.445

**Table 5:** Sums of squares for univariate ANOVA models—exercise condition (eg, single leg stance on ground, eyes closed; single leg stance on foam; single leg stance on foam, eyes closed) and residual—at each body location (*P*<.001 for all locations).

Leg-Body Position	Sum of Squares Exercise	Sum of Squares Residual
Right leg-torso	7.527	19.605
Left leg-torso	9.676	18.576
Right leg-knee	3.510	6.572
Left leg-knee	3.122	7.047
Right leg-ankle	5.337	4.795
Left leg-ankle	2.949	3.505

## Discussion

### Principle Results

There are 2 unique contributions that this study makes in the area of balance research. Firstly, we have demonstrated that a readily available form of software, namely a mobile phone app, can be utilized to quantify standing balance and can differentiate between exercises of varying difficulty. Our study concurs with other balance research that indicates poor balance on a foam surface as compared to a firm surface and with the eyes closed compared to eyes open [6,7,9].

Second, we have shown that the optimal location for measuring standing balance using a low-cost accelerometer is at the knee, as opposed to the trunk, as suggested by previous researchers [14-17]. The majority of researchers who have utilized accelerometers have placed them at the approximate center of mass of the individual over the L3-4 lumbar spinous process [14-17]. This would measure trunk acceleration while performing balance exercises. Unfortunately, unless the individual’s balance is challenged significantly or the accelerometer has a high sampling rate, there can be little to no trunk movement detected by an accelerometer. Since our population was relatively young, healthy, and athletic and our accelerometer had a low sampling rate of 14-15Hz, we assumed that measuring at the trunk would not produce robust results for the easier balance conditions. Thus, we decided to measure at the trunk to stay consistent with previous research, at the knee as a midpoint between the ankle and the trunk, and the ankle since we assumed that the accelerometer would obtain the most robust measures due to the degree of perturbations around the ankle. Previous researchers have utilized accelerometers that sample at between 40-2000 times per second (40-2000Hz) [16,20,21]. Due to our low-cost accelerometer and low sampling rate, we wanted to determine the optimal placement of the mobile phone to obtain meaningful measurements of the subjects’ balance. It is interesting to note that most mobile phones released after 2013 have sampling rates of at least 150-200 times per second [22]. This seems to suggest that as mobile phone technology advances and the embedded accelerometers improve, myAnkle may be able to provide more robust measures of standing balance. Of note, for more difficult exercises, the ankle location may provide greater detection sensitivity but this would be at the cost of potentially greater asymmetry between the left and right legs. A recent study evaluating a mobile application for home monitoring of Parkinson’s disease also utilized a mobile device strapped to the patient’s ankle to conduct a walking and turning test [23]. In terms of ease of application and feasibility for patients, the ankle may be the preferred location for self-monitoring of balance and gait.

The degree of difficulty of all 8 balance conditions, as determined by independent clinician rankings, showed high agreement with the ranking results of myAnkle. This is suggestive of strong validity for the application when compared with clinical judgement.

### Comparison with Prior Work

Accelerometers are utilized in research and clinical settings to measure standing balance and gait patterns; compare injured versus uninjured subjects and older fallers versus nonfallers; test balance abilities of patient’s with Parkinson’s disease; and classify human movement and sport performance [14-17,20,21,23,24]. Previous research has established that balance measurements performed using research-grade accelerometers show concurrent validity when compared to both force plates, clinical balance tests, clinician raters and they also demonstrate test-retest reliability[14-17,20,24]. Based on previous studies, it is clear that the use of an accelerometer to measure balance is not a novel concept. What is novel about this study is the use of a mobile phone app to quantify standing balance. Five previous studies have utilized a mobile phone to quantify human movement in the context of rehabilitation. The first utilized a mobile phone to classify different sporting activities, the second to assist with Gaze Stabilization Exercises, and the third to test the validity of its measurements to the Timed Up and Go test [25-27]. The 2 most recent publications also utilize a mobile device’s inertial sensors to perform home monitoring of frail elderly and to measure aspects of gait and turning in subjects with Parkinson’s disease [23,28].

Mobile phones and apps are becoming ubiquitous throughout the world. In fact, it is estimated that in 2012, of those who own a mobile device over 40% of European adults and 50% of US adults owned a smartphone [29]. On a global scale, it is projected that smartphone users will reach 2 billion by 2016 [30,31]. This widespread use of mobile phones and smartphones has led to the emergence of mobile health or mHealth.

The field of mHealth has emerged as a way for patients to monitor their own health and for health care providers to monitor and provide remote assessment and treatment for their patients—especially for those in resource-constrained areas [31]. The potential benefits of mHealth include being remote, patient-centered, and cost effective, as well as the ability to improve research and health outcomes, reduce health care visits, allow patients to self-manage chronic disease, and empower patients with information and to measure body functions that previously required specialized equipment and/or a health care provider [32,33]. The pace of mHealth development and uptake is moving very fast; however, one of the main critiques of mHealth is that growth has far outpaced the outcomes and research that validates its use as a health care tool [31,32]. An mHealth app review in October 2013 stated that there are currently 43,000 health care apps available for download from the US Apple iTunes app store, but the majority only provide information and have very simple and limited functionality [34]. Researchers are recommending increased input from health care providers and patients for mHealth initiatives, as well as more rigorous evidence-based research in the field of mHealth to demonstrate its efficacy as a health care tool [31-33].

With the emergence and popularity of mHealth, the current paper suggests a method of measuring standing balance that takes advantage of the capabilities of a mobile phone while addressing some of the recommendations and limitations of mHealth identified in the literature. The myAnkle app can bridge the gap between how balance is measured by the clinician, the researcher, and the patient. It can empower patients to understand and measure their standing balance and, in the future, improve compliance with their rehabilitation for balance deficits. The myAnkle app can also provide the clinician with a method of assessing and tracking standing balance that is easy to perform in the clinical setting without expensive equipment or additional training.

A key aim of this app is to make balance measurement as widely accessible and usable as possible. The mobile phones utilized were purchased for $160 CAD each, and the armband can be obtained online from $5 to $30 each. One of the initial barriers to the widespread use of this app was the requirement of offline post-processing of the data. However, the latest version of myAnkle, which can be downloaded for free from the Google Play Store, performs all data processing in real-time without the need for post-processing.

### Study Limitations

The main limitation of this study is that myAnkle’s balance measurement was not compared to a gold standard such as a force plate or a research grade accelerometer that was tested for validity and reliability. The measure was also not compared to a visual scale, which could enhance its clinical applicability. The next phase of research and testing of myAnkle will involve testing the app concurrently with a force plate and a visual scale to determine concurrent validity and reliability. We also plan to test the app on multiple mobile phones to determine if the app is reliable when used concurrently on different devices.

### Conclusions

The myAnkle app measured significantly greater mean acceleration values for balance conditions of greater difficulty. The myAnkle app also showed foam surface and eyes closed balance exercises to be more challenging compared to the same exercises on the ground or with eyes open, with the knee location as the optimal site for measuring standing balance. The myAnkle app demonstrated strong validity as compared with expert clinical ratings. These results indicate that myAnkle may have wider utility as a measurement tool for standing balance in clinical, research, and home settings.

## Multimedia Appendix 1

Screenshot of the main screen.

## Multimedia Appendix 2

Screenshot of the exercise selection screen.

## Multimedia Appendix 3

Screenshot of the exercise instruction screen.

## Multimedia Appendix 4

Screenshot of the exercise results screen.

## Multimedia Appendix 5

Screenshot of the progress screen.

## Abbreviations

ANOVA: analysis of variance
BESS: Balance Error Scoring System
C: correction factor
DL: double leg on ground, feet apart
FDL: double leg on foam, feet apart
FSLS: single leg stance on foam
FSLSE: single leg stance on foam, eyes closed
FT: double leg on ground, feet together
R: resultant vector
SLS: single leg stance on ground
SLSE: single leg stance on ground, eyes closed
TAN: tandem (1 foot in front of the other)
