# Supplementary figures and images for: Novel Use of a Smartphone to Measure Standing Balance
Source: JMIR Rehabil Assist Technol. 2016 Mar 29;3(1):e4. doi: 10.2196/rehab.4511 (PMC5454555; doi:10.2196/rehab.4511)

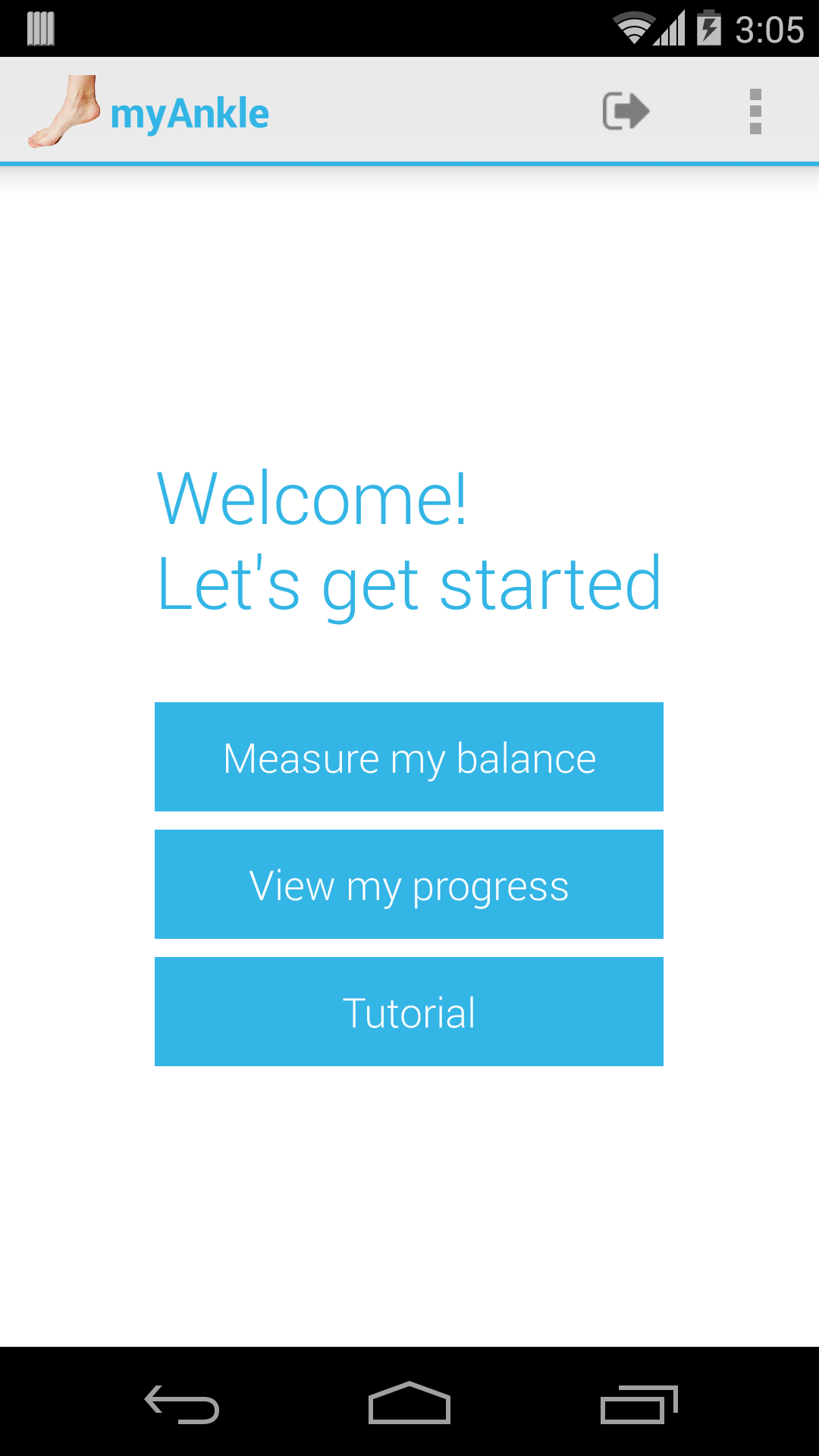

Supplement: Supplementary file 1 [file rehab_v3i1e4_app1.png]

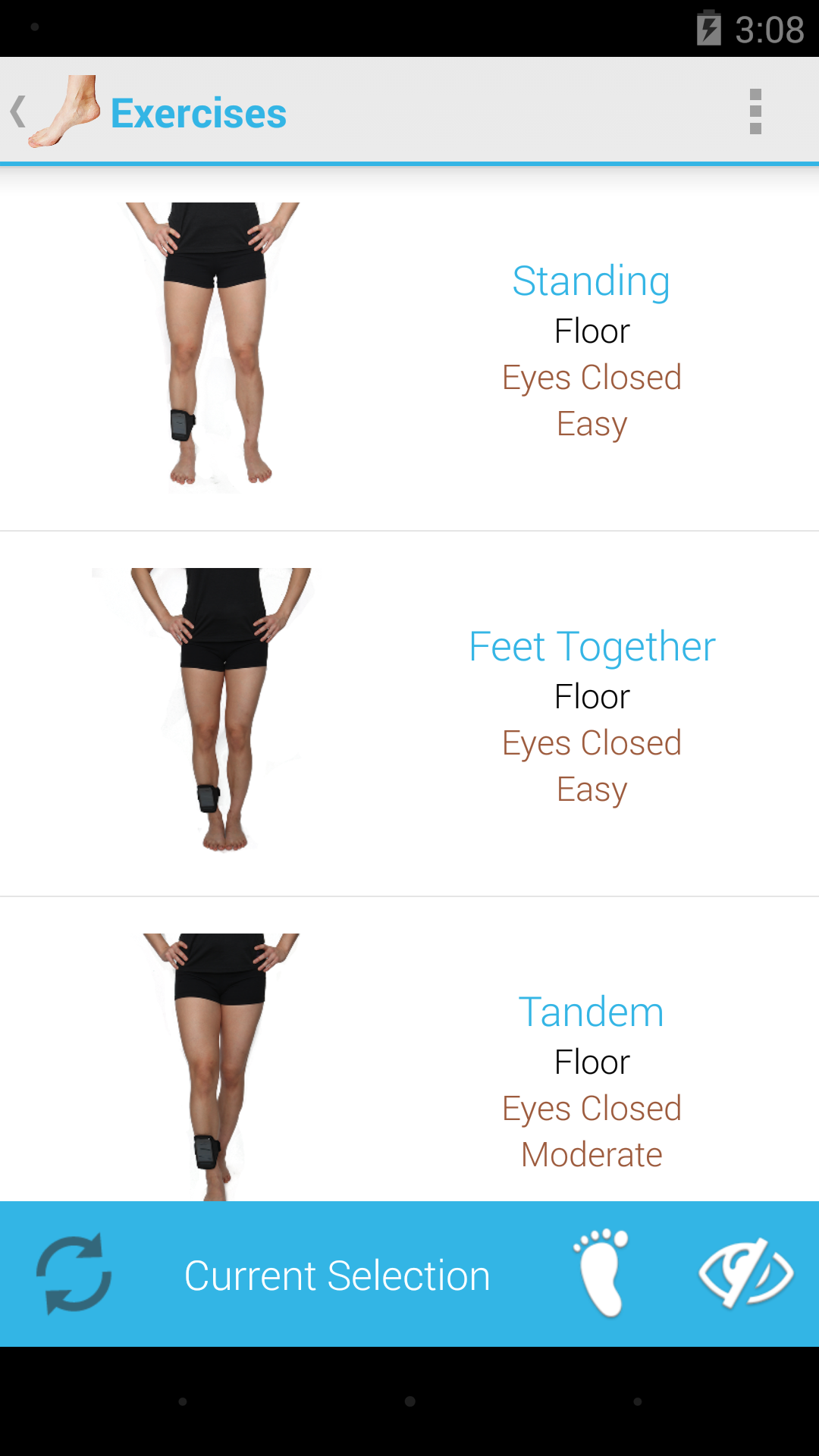

Supplement: Supplementary file 2 [file rehab_v3i1e4_app2.png]

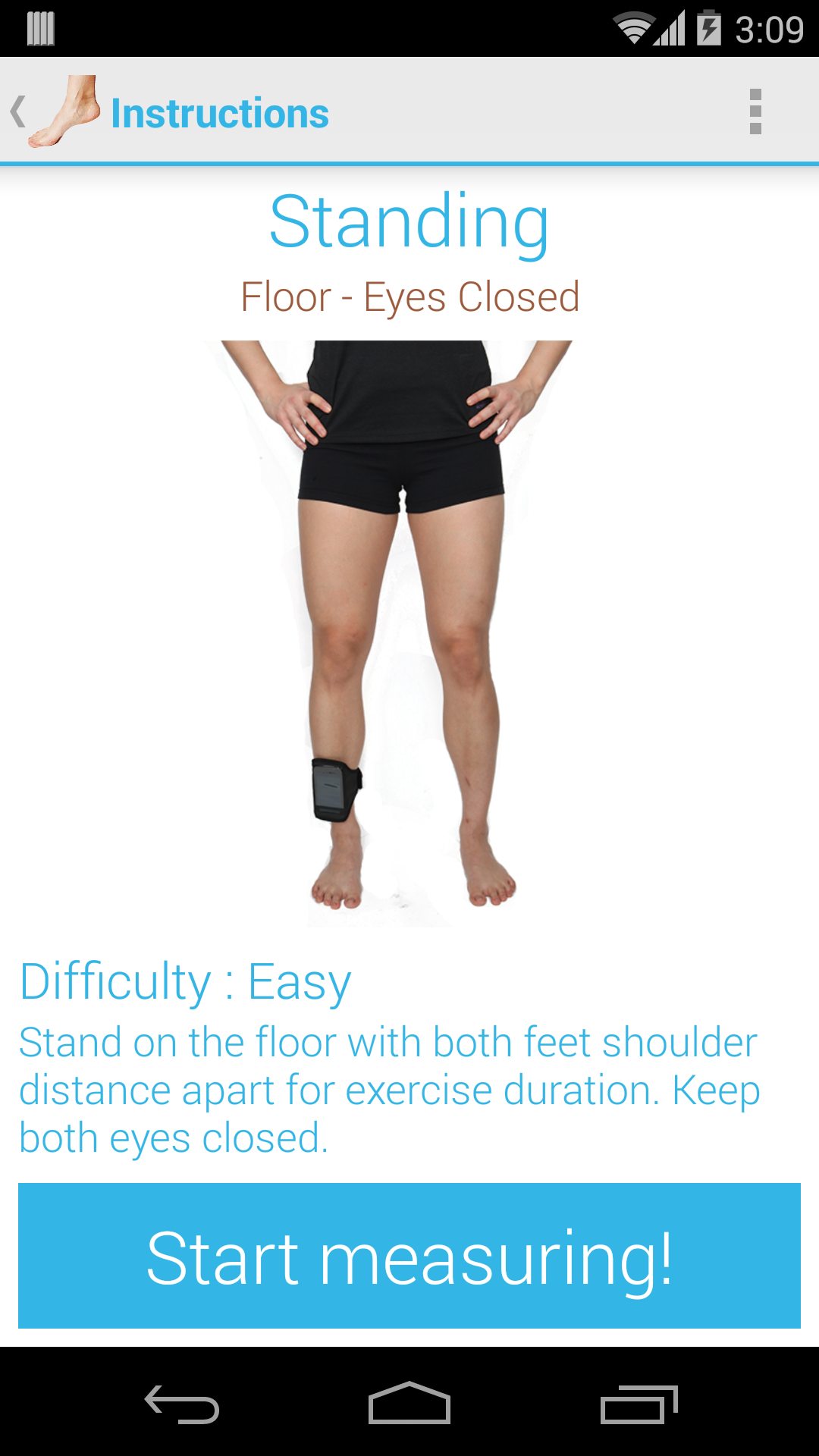

Supplement: Supplementary file 3 [file rehab_v3i1e4_app3.png]

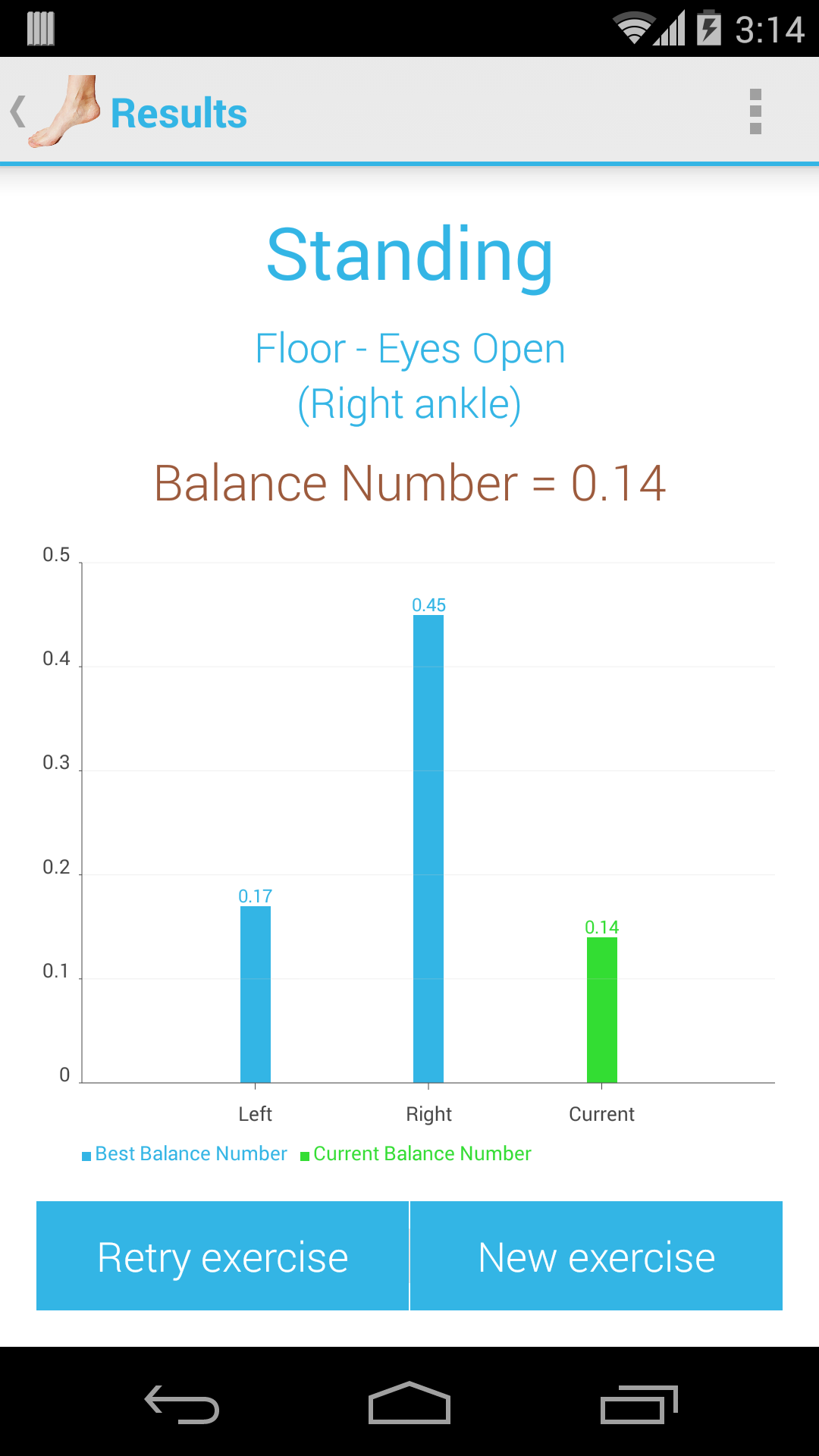

Supplement: Supplementary file 4 [file rehab_v3i1e4_app4.png]

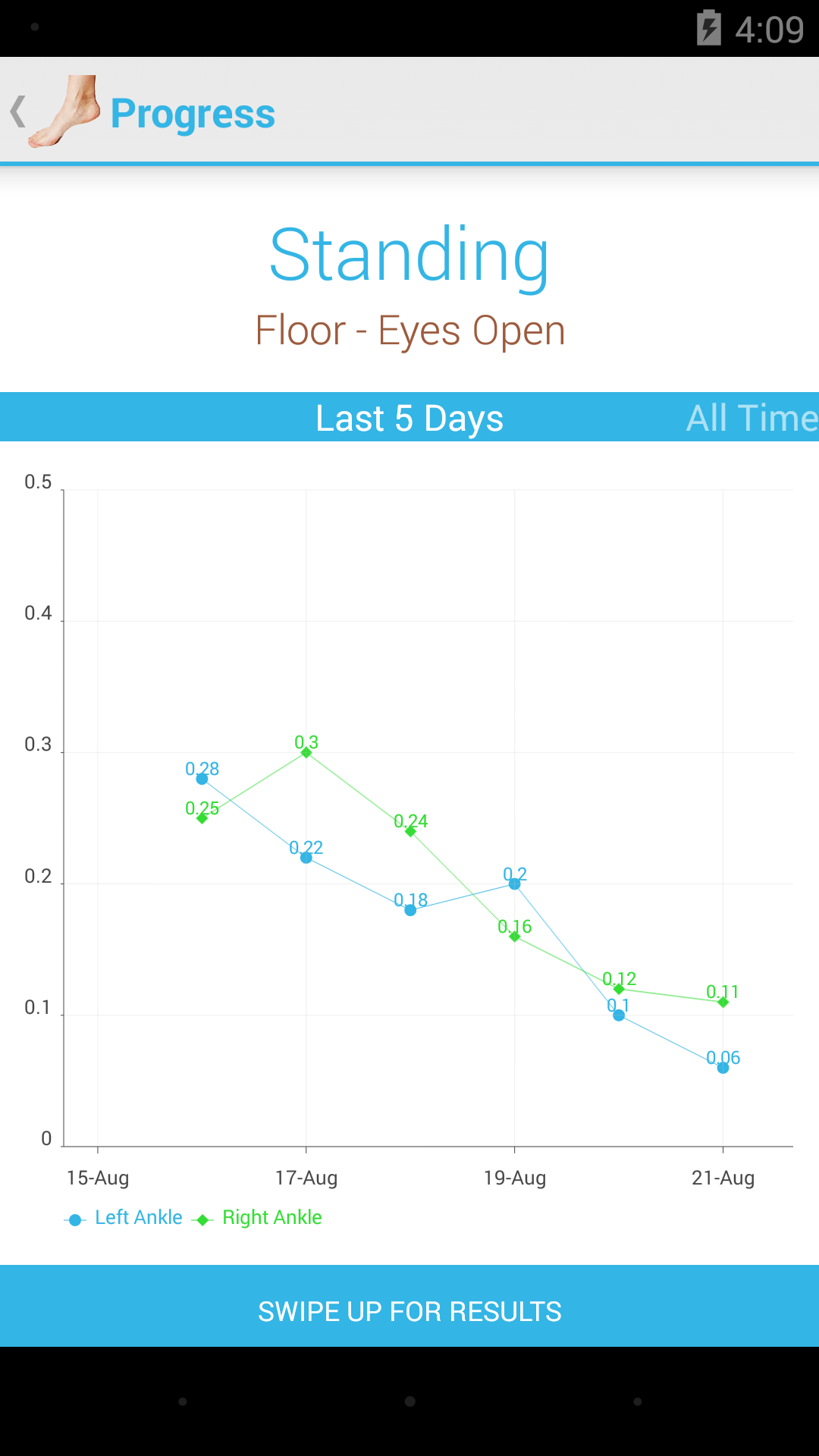

Supplement: Supplementary file 5 [file rehab_v3i1e4_app5.png]
